# Sawdust as a Byproduct of Wood Processing: Properties, Applications and a Reinforcing Filler in Hybrid Polymer Composites

**DOI:** 10.3390/polym17111523

**Published:** 2025-05-29

**Authors:** Tlholohelo Sylvia Sikhosana, Ntsoaki Joyce Malebo, Tladi Gideon Mofokeng, Mpho Phillip Motloung, Mokgaotsa Jonas Mochane

**Affiliations:** 1 Department of Life Sciences, Central University of Technology, Bloemfontein 9300, South Africa; tlholo0606@gmail.com (T.S.S.); njaymalebo@gmail.com (N.J.M.); 2 Centre for Nanostructures and Advanced Materials, DSI-CSIR Nanotechnology Innovation Centre, Council for Scientific and Industrial Research, Pretoria 0001, South Africa; tladimofokeng1988@gmail.com; 3 Department of Chemistry, College of Science, Engineering and Technology, University of South Africa, Florida Science Campus, Roodepoort, Johannesburg 1710, South Africa; motloung435@gmail.com

**Keywords:** natural fibre, wood-waste, sawdust, hybrid systems, polymer composites

## Abstract

There is a sizeable amount of sawdust produced from wood industries such as timber and furniture. In the past, sawdust has been utilized as a fuel source and in the manufacturing of furniture. Based on the limited use of sawdust, there is plenty of sawdust accessible from the industries. Sawdust is the material of choice due to its cost effectiveness, environmental friendliness, and biodegradability. However, if sawdust is not appropriately disposed or utilized better, it may have negative impact on the aquatic life and organic products. Hence, this review paper discusses the best possible methods or proper routes for the utilization of sawdust to benefit the environment, society, and the economy at large. Sawdust possesses superior capabilities as a reinforcing filler in various polymer matrices for advanced applications. This paper provides an in-depth discussion on sawdust hybrid composites in comparison to other natural fibres hybrid composites. The applications of various sawdust hybrid polymer composites for specific systems are also mentioned. Furthermore, the morphology and preparation of the sawdust/polymer composites and/or sawdust hybrid polymers composites are also discussed since it is well known that the properties of the natural fibre composites are affected by the preparation method and the resultant morphology. Based on the above, the current paper also plays a critical role in providing more information about waste to value added products.

## 1. Introduction

The demand for lightweight materials has prompted scientists to invest more research into natural fibre reinforced plastic matrices. The fabrication of natural fibre composites exhibited the possibility of enhancing the properties of the materials and reducing their ecological footprint. Currently, researchers have utilized agricultural, and forestry wastes such bagasse, rice husks, and sawdust (SD) as reinforcing fillers in various polymer matrices [1,2,3,4,5,6,7,8]. The reason behind the utilization of agricultural and forestry waste is due to the accumulation of industrial waste associated with the rapid increase in population and industrialization [9]. Wood-waste has emerged as one of the waste systems that requires serious attention in terms of recycling and effective utilization since it accumulates in various areas such as household activities, factories, and mills [9,10]. Globally, there is a slow progress for the effective recycling of wood waste [9]. According to statistics, the United States of America (USA) produces approximately 64.05 million tonnes/year of wood waste, with 25.76 million tonnes/year being non-recycled wood waste [11]. Furthermore, Pakistan and Australia produced 1.73 and 4.51 million tonnes of wood waste yearly, respectively [12,13]. In addition, the quantities of non-recycled wood waste were reported to be 346,189 (Pakistan) and 1.74 million tonnes/year (Australia) [12,13]. Wood is a renewable material that is useful in various applications such as paper, timber, and construction [14]. Chemically, wood consists of cellulose, hemicellulose, and lignin. Table 1 summarizes various residues produced in the processing of wood, with sawdust being the most dominant byproduct from various processing methods.

Sawdust produced from timber-based industries is used as a source of energy in manufacturing. However, the energy production process produces large amounts of wood-ash with unfavourable environmental impact [16]. Furthermore, wood ash is made up of particulate matter (e.g., carbon particles, heavy metals, polycyclic aromatic hydrocarbons (PAHs), etc.) which can easily cause air bore through winds which might present respiratory health complications. Based on the above concerns, there is a need to convert wood sawdust into valuable products. Sawdust has emerged as a potential reinforcing filler for various polymer matrices. Such composites are favoured because of good mechanical properties, positive environmental impact, and dimensional stability. Therefore, this review paper discusses an overview of bio-fibres, their applications, the history of sawdust, and a comparison of sawdust hybrid composites with other natural fibres hybrid systems. Furthermore, various sawdust polymer hybrid systems are also reported in relation to their applications. The morphology and preparation of the sawdust polymer and/or hybrids system are also discussed.

## 2. An Overview on Bio-Fibres

**Figure 1:**
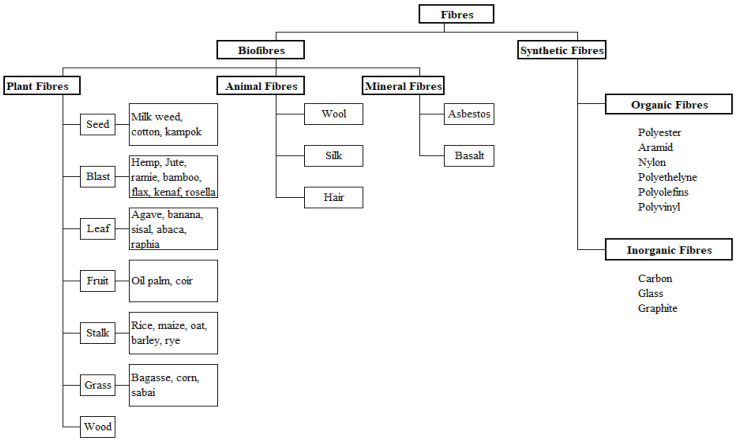
Classification of fibres, an adaptation from [28].

Fibres are substances distinguished by their flexibility, fineness, and high length-to-thickness ratio [17]. Based on their source, they are classified as natural, synthetic, or inorganic, as shown in Figure 1. Glass and carbon fibres are the most commonly used synthetic fibres, and glass fibres are the most widely used of all synthetic fibres because they offer excellent strength and durability, thermal stability, impact resistance, chemical, friction, and wear properties. Other synthetic fibres, such as aramid, basalt, polyacrylonitrile, and polyethylene terephthalate, among others, provide similar benefits but are rarely used due to their toxicity to both humans and the environment [18,19,20,21,22]. Although the utilization of synthetic fibres make good economic sense, safety concerns remain an issue, thus the interest in fibres obtainable from various biological sources such as plants (cotton, sisal, hemp, kenaf, jute, etc.) and animals (sheep, butterflies, spiders, etc.), that represent both economic and environmental sustainability, have become an option [23,24,25,26,27].

**Figure 2:**
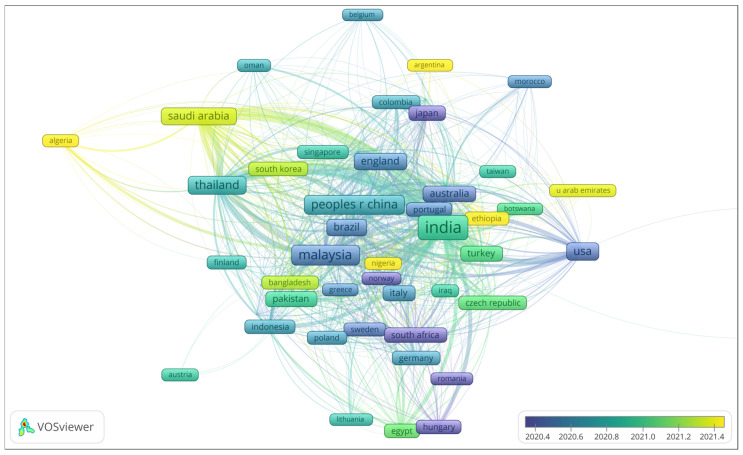
Selective leading countries in the field of natural fibres.

In recent years, synthetic fibres such as glass have dominated the fibre market [29]. However, concerns about the continuous use of toxic and non-renewable resources have led to a demand for sustainable materials, resulting in a renewed and growing interest in bio-fibres [30]. Stringent environmental regulations are forcing researchers across the globe to develop environmentally friendly materials, and fibres from natural resources offer significant properties with potential applications in modern industry [19]. Plant fibres, which are perceived as eco-friendly sustainable materials, are increasingly being used in a variety of markets, particularly as reinforcements and/or fillers in various composite matrices [30,31,32,33,34]. Figure 2 shows popular countries in the field of natural fibres. Asian countries, especially those in tropical regions (e.g., People’s Republic of China and India), lead in natural fibre production due to abundant resources such as jute, sisal, coir, and bamboo. Traditional knowledge and expertise in working with natural fibres have been passed down through generations, providing a basis for innovation and advancement [32].

**Figure 3:**
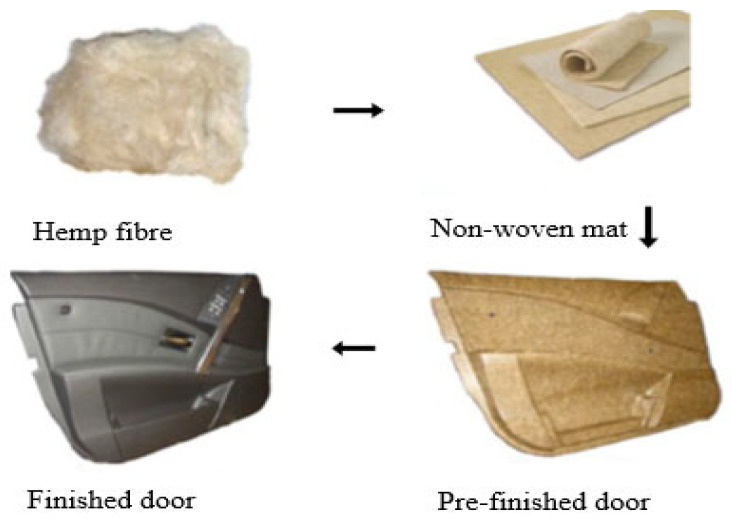
A typical procedure for fabrication of car inner door panels [48] (open access copyright MDPI).

The plant fibre market is now a lucrative enterprise with numerous applications across industries [35]. The automotive and construction industries are at the forefront of utilizing natural fibre-reinforced polymer composites, capitalizing on their distinct benefits to suit changing industry demands and consumer preferences. In the automotive industry, where lightweight and eco-friendly materials are urgently needed, natural fibre composites offer a compelling solution. Bio-fibre-based composites can drastically reduce weight, up to 30% in some cases, and provide substantial reductions of about 20% for automotive parts [8]. This weight reduction is crucial for improving fuel efficiency and reducing emissions, in line with strict environmental regulations and consumer preferences for environmentally friendly vehicles. In construction, similar demands for lightweight and sustainable materials exist, driving the adoption of natural fibre composites for applications such as roofing, insulation, and structural components [8,19,35]. The growing interest in bio-fibre applications is primarily motivated by the advantages of biodegradability and biocompatibility, abundance, cost-effectiveness, insulation, and tunability (feasible to modifications) [24,26,36]. Plant fibres have the lowest density of any structural fibre and possess higher specific strength and stiffness compared to glass fibres at a lower cost [37]. They offer the lowest possible carbon footprint due to fewer and less harmful emissions released during processing in comparison to other materials. Moreover, these fibres are generated by repeating monomeric units, consisting of carbon (C), oxygen (O), hydrogen (H), and nitrogen (N) [36,38,39,40]. Recyclability is another contributing factor to the sustainability of the fibres [24]. These fibres are not only easy to handle and work with, but they are also gentle on mixing and moulding equipment. The employment of bio-fibres as an eco-friendly and cost-effective alternative to synthetic fibres will aid in the preservation of ecological balance by reducing non-biodegradable waste through reuse while at the same time generating revenue [41,42,43,44]. The use of plant fibres can be traced back to prehistoric times when the Egyptians used them as reinforcing materials in bricks and the Babylonians used them for burial purposes [45]. Plant materials, such as paper reed, cotton, and linen fibres have been used throughout the existence of mankind to make clothing, paper, pottery, etc. To date, they remain the primary source of materials in textile, food, bioethanol production, building, and construction and automotive, among others [45,46,47]. The dawn of civilization saw scientist Hendry Ford develop an automobile body out of hemp fibres, which inspired Mercedes Benz and BMW to incorporate the same fibres into modern car components [45]. Figure 3 depicts a typical procedure for using bio-fibres to make car inner door panels for automobiles. A mat made entirely of bio-fibres, or a 50/50 blend of bio-fibres and any suitable polymer matrix can be produced. This mat can be moulded into any desired shape and laminated with plastic or leather film, or it can be adorned however desired [48]. By employing these materials, weight savings of 20 to 50% in interiors can be achieved, significantly increasing the environmental performance rating of automobiles [49].

Historically, plant fibres were predominantly employed as the main matrix, with thermoplastics serving as fillers in minor proportions [45,48,50]. However, recent research have explored alternative trends. These long, thin, and flexible strands of material can also be employed as both fillers and/or reinforcement in technological developments to improve the qualities of biodegradable polymers. Some of the most commonly used commercial fibres include flax, hemp, and kenaf, and it is often in conjunction with eco-friendly matrices such as polypropylene, epoxy, and polylactic acid (PLA), to name a few [30,31,51,52,53]. Most automakers now use bio-fibres in their creations to reduce vehicle weight, production costs, and environmental effects while leveraging these characteristics as a competitive marketing advantage [48,49]. Presented in Table 2 are the motor producers who adopted fibres from unconventional sources to produce interior car components. The different parts produced among others include inner door panels, seat covers and insulations etc.

Scientists often draw inspiration from nature and employ biological compounds as design cues for advanced materials. Nature provides bio-fibres with a wide range of shape, size, density, and microfibril angle. Plant fibres represent a class of macro-molecular biomass materials that can be further developed and widely used, thus the vast interest to incorporate them into composites materials. The molecular structure and functional motifs of natural materials can be reproduced in artificial materials to give a blueprint for a variety of functionalities. Besides cotton, all plant fibres have three basic components, i.e., lignin, cellulose, and hemicellulose, which are crucial for structural support and plant survival [57,58,59,60,61]. They range from linear to highly branched structures with versatile features that permit modifications. Bio-fibres can be easily processed using a variety of green production techniques, including melt moulding, freeze-drying, phase separation, gas foaming or high-pressure processing, electrospinning, and/or a combination of some of these techniques. The usage of organic fillers that meet the majority of material design and development requirements to substitute synthetic fillers could result in both economic and sustainable development [62,63,64]. Cellulose is the major plant polysaccharide, followed by hemicellulose and lignin, respectively [60]. It is a chiral molecule composed of glucose monomer units linked by 1–4 linkages. The cellulose polymer chain is stabilized by the terminal reducing and non-reducing sugar units. Due to strong hydrogen bonds between hydroxyl group networks that form the cellulose molecule, the fibres are crystalline, which is currently a significant area of research [65,66,67]. Cellulose has been widely used as a raw material to replace commercial plastics and/or as a filler to overcome limitations in other polymers, predominantly in the tissue engineering and packaging industries [60,68,69]. Furthermore, cellulose contains an inherent structural characteristic that enables the molecule to be modified to perform the desired function anywhere in the industry. Surface modification of cellulose allows for substitution hydroxyl functional groups with other functional groups. This chemical variability is especially important for the current global drive to replace non-biodegradable petroleum-based commodities with more environmentally friendly products. As a result, cellulose has found use in water purification, fibre, membrane, film, and sensor production in the form of nanofibrous membranes, in addition to other industrial uses [69,70,71]. According to Baillie et al. [72], and Gregory et al. [73], hemicellulose is a branched polysaccharide composed of sugar monomers such as glucose, xylose, mannose, galactose, and arabinose, as well as uronic acids. Its constituents are known as “cross-linking glucans” because they can establish hydrogen bonds with cellulose and lignin. Unlike cellulose, they do not form microfibrils. Hemicellulose has a more open structure, several side branch groups, and is non-crystalline. Carboxymethyl, acylated, and cationic hemicelluloses, which contain a lot of free hydroxyl groups, are good candidates for chemical modification via cross-linking, esterification, etherification, and other methods. In reality, modified hemicellulose has outstanding water resistance, thermoplastic, and other properties. Chemically modified hemicelluloses could be used to create materials with distinct properties, increasing the value of biopolymers. These reactions alter characteristics of hemicellulose, allowing it to be employed in a range of applications such as medicine, films, hydrogels, and conductive polymers. Recent developments include food packaging due to functional properties like tensile strength and barrier properties which can be improved [74,75,76,77,78]. Lignin is a biomacromolecule with a cross-linked polyphenol and numerous functional groups that provide multiple crosslinking sites with other compounds. The three-dimensional network structure of lignin allows for significant chemical modification [79]. Due to the presence of phenolic, hydroxyl, and methyl groups, lignin is an excellent candidate for the development of new materials. Pure lignin contains phenolic hydroxyl groups (PhOH) that can deprotonate and adsorb numerous metal ions, whereas kraft lignin (modified version) contains carboxyl groups (-COOH) that can also ion-exchange with metal ions as a result of the oxidative bleaching process. Due to these unique properties, lignin can be directly used as a binder, natural oxidant, ultraviolet (UV) protectant, and decontaminant [80,81,82,83,84]. Thus, there are numerous reports on the development of its blends for (i) heavy metal polluted water decontamination through cation exchange [80]; (ii) preparation of UV based antimicrobial food packaging composites using lignin as a reducing agent and a stabilizer [81]; (iii) and novel flame retardants with concurrent excellent smoke-suppression properties among others [82]. Typically, plant fibres are found in nature as composites of cellulose and hemicellulose embedded in a lignin matrix, with varied proportions of the readily identifiable constituent [28,36]. The cellulose fibrils are aligned along the length of the fibre, resulting in maximum tensile, flexural, and rigidity. Weak van der Waals and hydrogen bonds hold these components together. The cellulose molecules are hydrogen-bonded to hemicellulose. The cellulose-hemicellulose structure is then strengthened by lignin covalently linked to hemicellulose. The hydrophobic lignin fills voids in the cellulose–hemicellulose network as a coupling agent. Lignin stiffens the cellulose–hemicellulose network, protecting cellulose fibres from biological attack and environmental stress. While lignin and cellulose regulate water intake, hemicellulose influences the thermal and recyclable properties of plant fibre [23,85,86]. These biological cues are commonly employed as a guide for creating new materials in modern societies [57,87]. The basic components have a considerable influence on the characteristics of lignocellulosic fibres. Because of their filler and/or reinforcement capabilities, these components have been employed both individually and as a unit in a variety of matrices [85,88]. One of the major problems that researchers have had to deal with is the hydrophilicity of the fibres [32,89,90]. However, if cellulose and hemicellulose are generally occluded or capsuled by hydrophobic lignin, this could render the fibre biscuit hydrophobic because the other components are unavailable to establish interactions unless studied in the absence of lignin [91]. Nonetheless, because of the complexity of the structures some constituents, especially lignin, this concept is not fully understood. Although the primary purpose has been to remove lignin and hemicellulose for better results, some authors believe the combined effect has better prospects [28].

## 3. Sawdust History

The production of bio-fibre-based commodities came to a near-halt with the introduction of synthetic fibres in the 1940s [92]. However, critical conversations about natural resource preservation and recycling in the 1990s sparked renewed interest in renewable materials [93]. Recent developments in environmental consciousness have piqued their research interest, and their significance has grown [94]. Various bio-fibres with significant commercial value have been gracing the same markets as glass fibres. Kenaf, sisal, and hemp are a few examples of such bio-fibres. Wood fibres are not new but their popularity has increased in recent years, putting them on the list of emerging fibres alongside coir, *hibiscus*, and *bagasse* [95]. Like other important fibres, sawdust does not only offer low-cost and less denser products. In many cases, improved mechanical properties suited for engineering applications are achieved in the presence of sawdust [52,94,96,97,98]. Another intriguing attribute to consider is the biodegradability of sawdust. Although its decomposition depends on variables like wood type, composition, environmental conditions, etc., sawdust can generally break down within six months of soil exposure [99]. When combined with bioplastics, the composites can degrade within 6 to 12 months. This rapid degradation, in contrast to the decades-long persistence of traditional plastics, renders them well-suited for circular material use and aligns with current sustainability efforts [100].

Sawdust is a versatile, low-cost, and easily accessible lignocellulosic bio-waste. It is a tiny, powdery wood debris generated by sawing and sanding in lumber mills and other related businesses. Sawdust is a byproduct of timber processes such as cutting, sizing, re- sizing, edging, trimming, and smoothing [101,102]. Vegetation produces thousands of tons of energy-rich lignocellulosic materials daily, which offer enormous potential for the long-term synthesis of chemicals and fuels [103]. Despite its widespread use as animal feed and in the creation of biofertilizers, sawdust management remains a challenge with prevalent esthetic consequences, especially in developing countries. The forestry industry is the largest producer of copious sawdust [104]. Typically, every 100 kg of wood treated in a sawmill yields 12–25 kg of sawdust. Softwood generally produces less residues or sawdust than hardwood, and the interior wood parts usually result in less sawdust than the leaves and bark. On average, wood sawing results in 12–25% dust [105].

**Figure 4:**
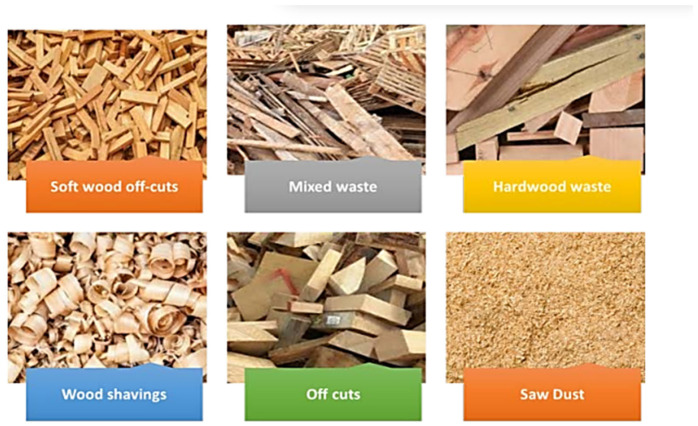
Different wood waste with permission from [108] (open access copyright MDPI).

Material wastage is a common phenomenon in many machining and manufacturing activities, making lumber waste management challenging. Most wood enterprises exist and aim to earn profits at the lowest possible cost, but waste extraction and disposal create additional expenses [106,107]. Wood waste refers to mechanically shredded wood residues, which include sawdust, hog fuel, bark, chips, slabs, shavings, trimmings, and mill ends, among others (see, Figure 4) [108]. Offcuts and chips are frequently utilized in commercial sawmills, as well as by workers and communities, to generate steam for kiln drying and fuel. The bark and shavings are mostly used as beds or bedding in necessaries and animal farms. Shavings and sawdust, on the other hand, are the most underutilized wood waste parts. Approximately 20–70% of sawdust along with some shavings and chips have to be disposed of, if not mixed with chips, for biofuel. Due to a lack of suitable disposal options, the majority of this waste is routinely discharged into the environment without treatment. Common disposal methods include burning, heaping at mill margins, and disposal alongside roadways and bodies of water [36,109,110,111,112].

Waste burning, in open fires or through incineration, emits pollutants into the atmosphere, which can impair both human health and the environment [113]. Incinerating sawdust releases noxious fumes and gases, including sulphur oxides (SOx), carbon monoxide (CO), nitrogen oxides (NOx), volatile organic compounds (VOCs), and particulate emissions, depending on its source and composition. In addition, the ash residue left after burning may be harmful if not handled and disposed of correctly. Nitrogen and sulphur oxides constitute substantial portions of emissions. These gases contribute to the formation of photochemical smog, an atmospheric phenomenon defined by a complex mixture of pollutants that not only degrades air quality but also actively depletes natural ozone levels in the atmosphere, worsening environmental issues. Generally, burning of biowaste waste leads to acidification and eutrophication. These pollutants can cause acid rain and harm the surrounding ecosystems by increasing soil and water acidity levels. This can have detrimental effects on both terrestrial and aquatic habitats. Additionally, the ash and particulate matter from incineration can contribute to nutrient overload in water bodies, further exacerbating eutrophication and disrupting ecological balances [113,114,115,116]. 

**Figure 5:**
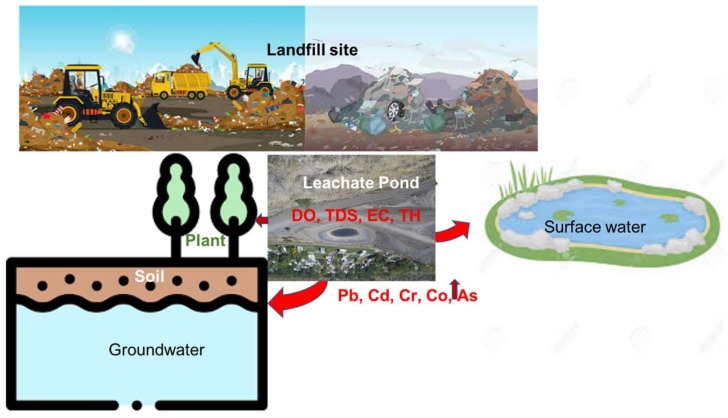
Effects of landfill leachate on the surrounding eco-systems [114,115,116,117,118,119] (open access copyright MDPI).

On the other hand, sawdust disposed of in formal and informal landfills is subject to chemical, physical, and biological reactions and transformations during which nutrients, elements, and gases are released. These come into contact with water and other undesirable substances to create leachate [117]. Leachate is defined as any contaminated liquid that is generated by percolating through a solid waste disposal site and moving into surface areas [118]. Leachate negatively impacts groundwater, surface water, soil, and plants, as indicated with red arrows in Figure 5. Landfill leachate alters the physicochemical parameters and heavy metal concentration in surface water, groundwater, soil, and plants. They exhibit biodegradable and non-biodegradable organic matter, compounds, humic substances, ammonia, nitrogen, heavy metals, and chlorinated salts. Improper landfill leachate management may have negative consequences for both the environment (biodiversity) and public health [119]. The potential impacts of landfilling sawdust are illustrated in Figure 5. This figure highlights key water quality indicators that can be affected by the leachate produced from sawdust decomposition in a landfill, including Dissolved Oxygen (DO), Total Dissolved Solids (TDS), Electrical Conductivity (EC), and Total Hardness (TH). Generally, improper handling and management of waste, including sawdust, pollutes the ecosystem and emits noxious fumes and gases into the atmosphere, thereby contributing to global warming and its emission back to humanity, resulting in environmental hazards. Hence, researchers are researching ways of converting sawdust into value-added goods to provide a green, clean, and sustainable environment while also contributing to the global waste management system [114,115,117,119].

## 4. General Applications of Sawdust

Traditionally, wood residues were considered to have little to no economic value byproducts. However, nowadays wood residues are treated as a valuable resource from which energy, fuel, and other profitable goods can be derived [120]. Sawdust, for example, is widely regarded as a polluting timber-industrial waste, but it has the potential to become a valuable commodity as a raw material in the manufacturing industries [108,111,112,121]. Sawdust is a low-cost energy source, an excellent adsorbent for extracting heavy metals and other impurities from water, and an inexpensive building material [101,122]. As such, the energy industry burns it directly or indirectly to produce wood gas, briquettes, pellets, and other products, whereas the building and construction sector uses it to manufacture wood boards, light construction materials such as shelves, notice boards, walls, and roof sheeting for mobile homes, and as an insulator in the refrigerating system and cold conservation [112,120]. Furthermore, as an absorbent, it has been widely utilized to contain or clean up surface oil spillages [123], and as an alternative point-of-entry (PoU) water treatment in areas where modern water treatment methods are out of reach for people [124].

**Figure 6:**
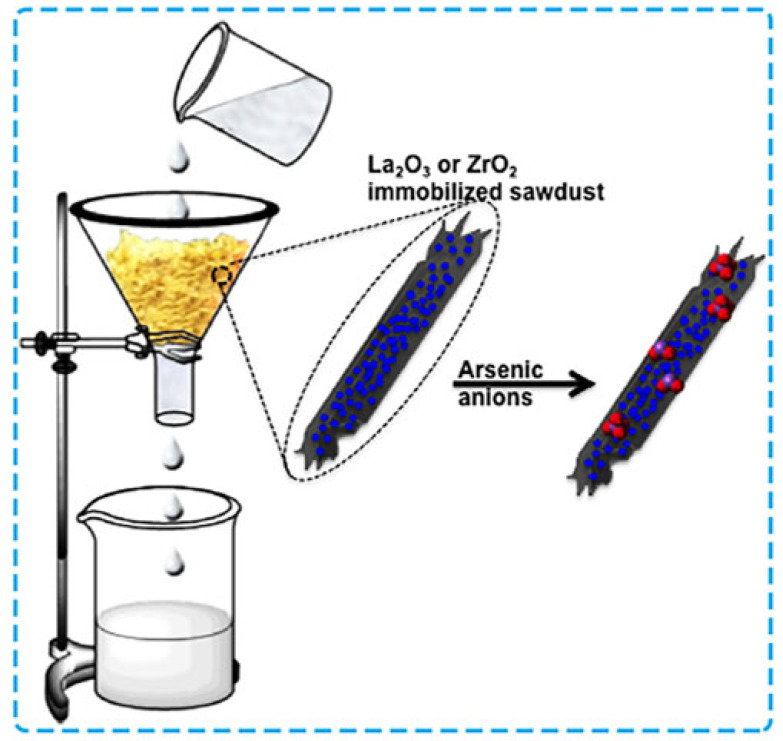
Removing arsenic pollutants with modified sawdust [101]. Copyrights American Chemical Society.

Sawdust is composed of various functional groups such as nitrogen (-N), oxygen (-O), sulphur (-S), and phosphorus (-P), conferring the ability for effective removal of environmental pollutants. These groups are essential in facilitating pollutant removal mechanisms like hydrogen bonding, where the polar groups on the sawdust surface interact with polar contaminants, and ion exchange, where positively charged metal ions in solution are exchanged with negatively charged functional groups on the sawdust surface, aiding in the purification of soil or water systems [101,125]. Setyono and Valiyaveettil [101] revealed that unmodified and modified sawdust can be used to remove impurities from wastewater. Figure 6 is an illustrative example of the removal of arsenic ions (As^3+^ and As^5+^) from aqueous solutions with renewable metal oxides (La_2_O_3_/ZrO_2_)-sawdust hybrid composites. The dominant adsorption mechanisms differed for both hybrids. Electrostatic interactions were found to be more prevalent for La_2_O_3_-sawdust, while ligand exchange was more common for ZrO_2_-sawdust. The ZrO_2_-sawdust composite exhibited extraction capacities of approximately 12 mg/g for As^5+^ and 29 mg/g for As^3+^, while La_2_O_3_-sawdust composites demonstrated higher capacities of about 22 mg/g for As^3+^ and 28 mg/g for As^5+^ [101]. Moreover, ZrO_2_ and sawdust composites were able to regenerate with an efficiency of approximately 50%, whereas La_2_O_3_ and sawdust could be fully reused without any loss in adsorption efficiency.

Besides making domestic fires and firing up power plants, due to its high cellulose content, sawdust can be used as a raw material in the production of bioethanol. Bioethanol is a clean, green, and renewable energy produced from lignocellulosic biorefinery that has attracted great attention to substitute petroleum and fossil fuels while relieving environmental and energy crises [126]. According to Amaefule et al. [127], bioethanol can be used as fuel on its own or combined with gasoline. Bioethanol, both in pure form and blended with gasoline, serves as a versatile fuel option, commonly used in flexible fuel vehicles in countries like Brazil and the United States. Blends such as E-10, E-15, and E-85 offer varying ethanol concentrations. Its use can replace gasoline additives, offering strong stopping power and other benefits like biodegradability, sulphur-free nature, and less hazardous byproducts from incomplete oxidation. As the most utilized biofuel globally, bioethanol finds applications not only in transportation but also in domestic cooking, fuel blending, hydrogen production, and as a precursor for various chemical products. In a two-part bioethanol production study [127], different particle sizes of sawdust (212 μm, 300 μm, and 500 μm) were preheated, hydrolysed with H_2_SO_4_, and fermented with two species of yeast, i.e., *Saccharomyces cerevisiae* and *Saccharomyces chevaleiri*. Distillation recovered bioethanol of varying weight, pH, density, viscosity, flash point, and heating value, with alcohol content consistently at 69%.

Sawdust is a durable and decay-resistant material that can serve as a long-lasting substitute for traditional building materials [98,128]. Cultrone et al. [129] investigated the effects of incorporating sawdust as an additive to clayey earth used in brick production. The sawdust, sieved to remove larger grains, is mixed in varying proportions (2.5, 5, and 10%) with the clay for experimentation. The bricks were handcrafted due to the demand for less standardized products in construction and restoration projects. Handmade bricks are preferred for their versatility in size, shape, and finish compared to industrially extruded ones. The manufacturing process involves moulding the clay/sawdust mixture, cutting it into cubes, and firing at temperatures ranging from 800 to 1100 °C. Slow cooling prevents cracking during the transition of quartz types. After firing, the bricks are submerged in water to eliminate CaO grains, which otherwise could lead to cracking due to lime blowing. The resulting bricks were lighter in weight with better thermal insulants, for which extensive vitrification was not required. This would enable them to be fired at lower temperatures, so reducing energy costs.

Sawdust comes in a variety of sizes and shapes to suit a wider range of industrial applications. A range of factors, including the source (wood type and geographical location) and manufacturing process, and influence on the physical and chemical qualities of sawdust. Its composition differs significantly depending on the type of processed tree species [122,130]. Table 3 summarizes the typical sawdust composition based on the source. The shape, dimension, and amount of dust produced are all influenced by the physical and chemical properties of the sawed and abraded wood, as well as the form, dimension, sharpness of cutting instruments, and technological circumstances of sawing and sand abrading operations [131]. Saw blades play a critical role in determining particle size, typically classed as oversized, coarse, pin, or fine particle-sized, with diameters of >850 m, 500–850 m, 400–500 m, and 177–400 m, respectively [132]. Sawdust has features similar to wood, but because it is in particle form, some of its structural properties are modified. However, sawdust can be reformed into wood and imitate it in some applications when necessary. When sawdust is used as an energy source, it can produce the same amount of heat as other fuels, making it an adequate wood substitute. Sawdust is a versatile material that can be moulded into different shapes and sizes using various techniques, making it an ideal material for advanced designs [120,124].

Research trends have shown that sawdust and its derivatives can be used as alternatives in the production of sustainable engineering materials for a variety of applications [133,134,135,136,137,138], ideally as reinforcing materials in inferior bioplastics to provide the requisite superior physical and mechanical properties for a wide range of technical applications, while also addressing the issues of depleting fuel supplies and environmental degradation [139,140,141,142,143]. Sawdust has been predominantly used in building and construction as a filler for more than four decades [98]. Besides substituting sand in concrete, other sawdust composites include particleboard, floor slabs, panels, attic, and brick. Introducing sawdust as an intriguing biological waste and transforming it into a resource for sustainable remediation technologies might increase its application spectrum in varied applications [101,102].

## 5. A Brief Discussion on Hybrid Systems vs. Sawdust Hybrid Systems

**Figure 7:**
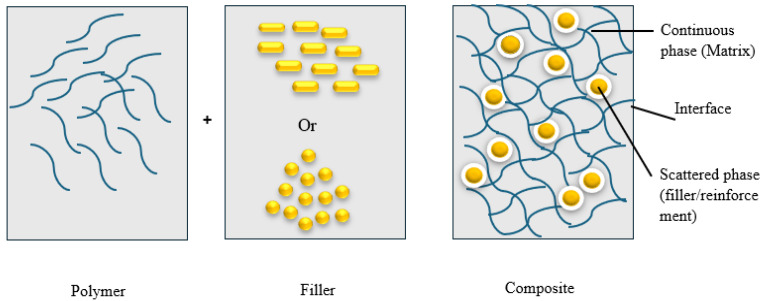
Arrangement of both matrix and filler through interface of the composite.

The term composite is Latin derived, composĭtus, used to describe a multi-phase combination material made up of two or more materials with significantly diverse chemical and physical properties through an interface [144]. The composite material not only retains the main properties of the original materials but also possesses new properties that none of the original components exhibit [58,88,145,146]. The two most commonly mentioned composite phases are the matrix and the filler. However, an additional transitional phase called the interface exists between the phases. The arrangement of both matrix and filler through interface of the composites is illustrated in Figure 7. During composite formation, the matrix is represented by a continuous, homogeneous and/or isotropic phase, with a lower modulus and high elasticity (such as polymers extracted from plants) polymers, carbon, ceramics, or metals. The filler, which is usually in the form of short or long fibres with much higher physical and mechanical qualities, is surrounded by the matrix. Generally, the scattered phase is stronger than the continuous phase, and it is responsible for enhancing one or more properties of the matrix [146,147,148].

The range of prospective engineering material requirements is diverse and particular and almost impossible to meet with a single material [149]. Combining two or more materials with distinct properties to create a composite material leads to improved properties than the individual components. Several natural and synthetic additives have been incorporated in various matrices to create novel inorganic and/or organic combination composites suitable for different engineering applications [145,150]. The different materials work together to give the composite unique properties. Generally, the composite material will have better and/or improved qualities than any of the components alone. Thus, composite materials have superior mechanical and physical properties, making them more suitable for a wide range of applications than individual composite components [151,152,153]. They are also versatile with unique characteristics such as high strength and modulus-to-weight ratio, ease of processing, and cost-effectiveness. As such, they are in high demand for practical applications in major industries, including aerospace, automobile, thermal and acoustic insulation, packaging, sports, defence, construction works, etc. [31,154]. One of the most significant advancements in material history has been the creation of composite materials and related design and manufacturing processes. The emphasis in engineering and research has turned away from monolithic materials and toward natural and/or synthetic fibre-reinforced materials. The fundamental advantage of using composite materials in advanced applications over single materials is that a composite is a multiphase material made up of two or more physically separate and mechanically separable components. Furthermore, composites are made by carefully mixing the ingredients to ensure homogeneous dispersion, resulting in perfect qualities [155,156,157]. Finally, the mechanical properties of composites outperform those of individual components, and in some situations, are distinct from constituent properties [158]. In recent years, natural fibres attracted research interest from relevant stakeholders as alternative reinforcing materials in polymer composites over conventional glass and carbon fibres. Natural fibres exhibit superior mechanical properties such as flexibility, stiffness, and modulus to glass fibres. However, despite natural fibres offering several attractive features as potential substitutes in composites, they are still relatively inferior to synthetic fibres. This limits the use of natural fibre-based composites in certain advanced applications [24,25,26,27,159]. Despite the fact that fibre-reinforced composites have acquired market share in advanced applications, the key limiting factors that impede the use of natural fibres in composite materials are low mechanical characteristics and water absorption. Bio-fibres, particularly plant-based fibres, absorb a lot of moisture, resulting in composites with low mechanical strength [160,161]. A possible remediation technique for improving the characteristics of fibre composites is hybridization, which is the outcome of a combination of many phases in which at least two varying additives are combined with the matrix. By integrating two or more fibre types, these hybrid composites provide a more superior combination of physical and mechanical qualities than non-hybrid composites. The three basic types of hybrid composites are synthetic/synthetic, synthetic/natural, and natural/natural hybridization. Synthetic/natural fibre-reinforced hybrid composites are the most commonly investigated hybrid composites due to their superior mechanical performance. Current trends, on the other hand, favour natural/natural hybridization, owing to the necessity to achieve a balance between environmental friendliness and mechanical strength. Through hybridization, the synergy of merging the particular benefits of each reinforcement and/or filler, general traits, and overall performance of the final composite can be improved [162,163,164,165,166]. The physical and mechanical properties of forestry and agricultural biowaste are diverse and vary greatly based on the origins and compositions of organic materials, ranging from soft to pliable plant matter [14,167]. Various factors, such as moisture content, cellular structure, and decomposition stage, affect the properties of biological waste. These traits have recently inspired some researchers to develop hybrid composites by combining multiple forms of bio-fibre. The hybrid approach maximizes the potential of both additions and in some cases, a synergistic impact may emerge [162,163,164,165,166]. Sawdust fibre hybrid systems are currently among the most researched natural fibre hybrid systems. In addition to their superior flexibility, stiffness, and modulus comparable to glass fibres, sawdust fibres are eco-friendly and relatively cheaper. Sawdust-improved systems are durable, making them ideal candidates for sophisticated engineering applications that demand high-strength materials with flexibility [139,140,141,142,143]. Traditional sawdust composite systems have shortfalls that limit their applicability; nevertheless, paring sawdust with other complementary fibres in selected matrices guarantees improved properties and hence viability in various applications [8,168]. While the hybrid system can use multiple fibres, a combination of only two types of fibre would be most effective. In principle, the hybridization of sawdust composites enables a balance between composite performance and cost unattainable with a single fibre [169]. Sawdust composite can be moulded into different shapes and sizes using various processing techniques, making it a versatile engineering material. The resulting composites are lightweight and easy to work with, therefore ideal for use in applications where weight is a consideration [52,98]. Over the years, researchers have evaluated the performance of numerous hybrid systems comprising sawdust and other natural fibres. A summary of selected studies and their findings are shown in Table 4. The hybrid composites in Table 4 have demonstrated outstanding qualities when the fibre is paired with their counterpart or another reinforcement(s). Overall, properties such as mechanical (tensile, flexural, impact strength, etc.), thermal, dimensional stability, morphological features, and moisture absorption were improved. In addition, the hybrids can be suitable for building and construction, textile, automotive, and packaging among other applications [52,98,139,140]. 

## 6. Preparation and Morphology of Sawdust Composites and/or Hybrids

Sawdust composites are made by combining sawdust with a binder or matrix material to create a composite with enhanced properties. Properties of these composites can be further advanced through hybridization with additional materials such as fibres, reinforcements, or nanoparticles [177,178]. The production method typically involves mixing the sawdust with the binder or matrix using various techniques such as compression moulding, extrusion, resin transfer moulding, thermoforming moulding or injection moulding, depending on the desired shape and properties of the completed product [52,170,171,175]. In compression moulding, for example, the mixture of sawdust and binder is placed into a mould and subjected to heat and pressure to combine the composite [179]. During the preparation, additional additives such as plasticizers, coupling agents, or fillers can be incorporated to improve specific properties including flexibility, adhesion, or strength [180]. Many matrices, including polymers, can be used to create sawdust composites or hybrid systems, and the selection of the matrix depends on the desired qualities of the composite, the processing technique, and the intended application [181]. Thermosetting, thermoplastic, and biopolymer matrices are a few of the most popular types of polymer matrices currently explored for sawdust [182]. Thermosetting resins such as epoxy, phenolic, and polyester resins are the most widely employed for sawdust composites [177]. They undergo a curing process where they are initially liquid or semi-liquid and eventually harden due to a chemical crosslinking reaction. They offer high dimensional stability, excellent chemical resistance, and mechanical qualities [183]. Epoxy resins, in particular, are known for their great strength and adhesion [184]. Thermoplastic polymers such as polyethylene (PE), polypropylene (PP), polystyrene (PS), polyvinyl chloride (PVC), and polyethylene terephthalate (PET) are also popular choices for sawdust composites. Thermoplastics offer good toughness, flexibility, and ease of processing [185]. Bio-based polymers have gained popularity in the production of sawdust composites due to growing demand for environmentally friendly materials [186,187]. Polymers derived from natural resources, such as starch, cellulose, lignin, or soy protein, can be used as matrices. These bio-based polymers have several advantages, including biodegradability, renewable supply, and low environmental impact. As such, there is a growing interest in sawdust composites based on bio-based polymers such as polylactic acid (PLA), polyhydroxyalkanoates (PHA) and polybutylene succinate (PBS) that exhibit enhanced stiffness and impact resistance compared to pure plastics [52,188,189]. These composites can be applied in different industries, including automobile components, building materials (such as decking and fences), furniture, packaging materials, and consumer items [190,191]. The morphology of sawdust hybrid composites refers to the structure and arrangement of the components within the composite [168,191]. Generally, sawdust particles are spread throughout the binder matrix. The distribution and orientation of sawdust particles in the composites have a significant impact on the mechanical, thermal, and physical qualities of the finished product. For uniform characteristics, sawdust particles must be distributed evenly throughout the matrix. The physical properties of the sawdust particles influence the total composite strength, stiffness, and durability [162,163,165,168]. For instance, the shape and size of sawdust particles influence the mechanical characteristics and homogeneity of composites. Smaller particle sizes often result in better interfacial bonding between the matrix and sawdust particles, leading to improved mechanical strength and stiffness of the composite [191,192,193]. Also, sawdust is believed to possess a high moisture content, which influences composite processing and performance. Increased moisture levels in the composite mixture tend to impede flowability, producing processing difficulties. It can also induce dimensional instability and reduce the strength of the final product [32,89,90,194]. Various pre-treatment approaches, such as those shown in Table 5, can be used to modify the surface properties of sawdust and improve its compatibility. These treatments can improve the interfacial adhesion of sawdust and matrix, resulting in desirable mechanical characteristics and water resistance of composites [32,90,111].

Besides pre-treatments, overall optimization of processing techniques and parameters in the production of sawdust hybrids or composites is crucial for achieving desirable mechanical, thermal, and physical properties, thus ensuring efficient and sustainable use of sawdust as a renewable resource [199,200]. Processing conditions and techniques have a significant effect on the properties of sawdust composites and/or hybrid systems [201]. Certain elements, such as the degree of homogeneity of the fibre, wettability of the fibre, fibre length, fibre volume fraction, processing parameters, and fibre orientation, must be taken into account in order to generate high-quality sawdust fibre composites [200,201,202,203,204]. Table 6 summarizes some of the key factors and their impact.

## 7. Degradation of Sawdust-Reinforced Biocomposites

Disposing of plastics at the end of application remains a challenge as available dumping sites are approaching exhaustion. In addition, conventional plastics are resistant to degradation in different media such as soil, water, and composts [205]. Natural fibres are biodegradable, and this is one of main reasons they are incorporated in polymer matrices to impart biodegradability and hasten their degradation rates. Therefore, incorporating lignocellulosic waste materials such as sawdust could accelerate the degradation rates of various polymer matrices. Degradation of sawdust-reinforced polymer composites is controlled by factors such as hydrophilicity of the fibres, which determines water absorption of the composites; the source of sawdust also affects degradation of natural-fibre reinforced composites [206]. The material properties such as load-bearing properties tend to decrease as a result of degradation. Fakhrul and Islam [207] investigated the biodegradability of PP/sawdust (5%) and PP/wheat flour (5%) composites exposed to different degradation conditions including moist soil, water, salt solution, and normal environmental conditions (moisture, temperature, sunlight, and wind). The water absorption tests indicated higher water absorption by sawdust-reinforced PP than PP/wheat flour counterpart, and this was attributed to highly hydrophilic nature of sawdust compared to wheat flour. Hydrophilic materials with high water absorption rates are likely to degrade faster through hydrolysis and microbial attack. In this case, PP/sawdust composites showed faster degradation rates, which were also confirmed by greater reduction in tensile properties of exposed sawdust-based composite compared to PP/wheat flour composites. Haque and Islam [208] evaluated the degradability of two epoxy-based composites containing sawdust and jute fibre at 5 wt.%. The biodegradation of the composites was conducted by keeping the samples in the drainage for a period of one year. The composites demonstrated higher water absorption than neat epoxy sample, with jute-based composite showing higher absorption capacity than sawdust composite. After one year, the samples were brownish, with many cavities noticed in the composites. These changes were attributed to swelling of the fibres and microbial attack. The tensile strength declined after one year of drain water exposure by 40 and 57% in sawdust and jute fibre-based composites, respectively. This indicated that jute-fibre offered higher degradation rate than sawdust, and this was attributed to lower lignin content in jute, which rendered it more prone to microbial attack and degradation. Abdel-Hakim et al. [209] reported an increasing trend in weight loss of sawdust-reinforced expanded polystyrene (EPS) buried under soil for 90 days. The composites were prepared by incorporating sawdust at concentrations ranging from 20 to 80%. Neat EPS did not show any weight loss after 90 days of burial in soil. The percentage weight loss decreased with increasing the amount of sawdust in composites. For example, the weight loss reached 2 and 6.3% in the composite containing 20 and 80% of sawdust. Dhal et al. [210] developed composites of PLA/PCL reinforced with sawdust at concentrations of 10, 20, and 30%, and evaluated their compostability. The time taken to degrade 10% of the materials decreased with increasing loading of sawdust, while time taken to degrade 50% of the material decreased with up to 20% of sawdust and increased again at 30% filler. In addition, time taken to degrade 90% of the material was not significantly affected compared to neat blend matrices. Prachayawarakorn and Hanchana [211] buried the composites of modified starch reinforced with sawdust under soil and evaluated changes in the mechanical response of the buried specimen after four weeks. The assessed mechanical properties (modulus, stress at maximum load) declined drastically as a result of burial in soil. The observed decrease was attributed to increased water uptake of the composites in the presence of sawdust, which facilitated degradation of the composites and subsequent decrease in mechanical properties. Islam and Islam 2015 [212] prepared the injection-moulded composites based on modified sawdust and recycled polyethylene. The composites prepared with unmodified sawdust showed higher water uptake, whereas treated sawdust composites showed less water uptake and swelling, attributed to reduced hydrophilicity of sawdust. Consequently, though weight loss increased with number of burial days, the weight loss of untreated sawdust-based composites was higher. Incorporating sawdust as hydrophilic material into polymer matrices serves as a viable strategy to enhance their degradation rates.

## 8. Conclusions and Future Recommendations

There is an increasing demand globally to reduce waste by recycling and reusing byproducts of processing. The forests industry produces wood, and the byproduct of wood processing is sawdust. Sawdust is inexpensive, abundant, and a lignocellulosic compound which can be easily converted into reusable materials. Due to its biodegradability, eco-friendliness, and low weight, sawdust has been utilized as a reinforcing filler in various polymer matrices. The properties of the resultant sawdust polymer composites depend on the preparation method and sawdust distribution within the polymer matrix, which have significant impact on the mechanical, thermal, and physical qualities of the finished product. Furthermore, various factors are playing a key role in the morphology of the sawdust/polymer composites and/or the hybrid systems, such as the type of the polymer, content of the sawdust within the matrix, particle size of the sawdust, and synergy of sawdust with other natural fibres. The particle size of sawdust and other reinforcing materials in the composite can greatly impact the morphology, while smaller particle sizes often result in better matrix dispersion, and thus a more homogeneous morphology. In most cases, there seems to be a poor interfacial adhesion between the sawdust and polymer matrix, which affects the properties of the resultant composites. There are possible treatments for sawdust to improve its adhesion with polymers, such as alkaline, silane, and acetylation. A careful inspection of the comparison of sawdust with other natural fibre hybrid composites showed that the sawdust hybrid system can compete with the well-known hybrid composites. In all the hybrid systems, there is a common component for the purpose of comparison, for example, the common factor is one of the fibres and a polymer matrix. In most of the sawdust hybrid systems reported in these review paper, there is a significant improvement in the mechanical properties of the hybrid systems. Based on the improvements, one can conclude that the sawdust hybrid systems can compete with well-known natural fibre hybrid systems, whereby the sawdust hybrid systems may be used in applications such as helmets, car interiors, and aerospace. For future purposes, more of the sawdust hybrid “green composites” based on the biopolymers, such as polycaprolactone (PCL), polybutylene succinate (PBS), and polylactic acid (PLA), are needed since majority of the sawdust hybrid systems are fabricated from epoxy matrix.

## Figures and Tables

**Table 1:** Selective sources and various types of residues produced in wood processing. Reproduced from [15].

Sawmilling	Bark, Split Wood, Sawdust, Trimmings
Particleboard production	Sawdust, bark, sander dust, panel trim
Forest function	Needless, leaves, sawdust, branches
Plywood manufacturing	Sawdust, core, bark, panel trim

**Table 2:** Selective applications of natural fibres in automotives produced with information from [48,54,55,56].

	Door Panels	Seat Parks	Boot Liners	Spare Wheel Lining/Cover	Instrument panels	Insulation	Others
Renualt (Twingo, Clio)							Dashboard area including other rigid parts (not specified)
Ford (Mondeo, Focus, Ford fusion, Lincoln)	√	√	√				
Volkswagen (Golf, Passat, Bora)	√	√	√				Trunk lid
Audi (A2, A3, A4, A6, A8)	√	√	√	√			Hat holder/rack
Citron (C5)	√						
Mercedes-Benz (C, S, E, A class truck and Vision EQXX)		√					Bumper, wheel, box, roof cover, glove box, internal engine cover
General Motors (Chevrolet—Impala, Cadillac De Ville)		√	√		√		Back-shelf trim panels
BMW (3, 5, 7 and i3 series)	√	√	√		√	√	Foot well covers, dashboard
Fiat (Alfa Romeo 146, Brava, Punto, Marea)	√						
Opel-Astra	√						Hat racks and spare tyre covers
Daimler-Chrysler (A, C, E, S)	√						Dashboard, windshield, window pillar panel cover, business table
LOTUS (Eco Elise)	√	√					Spoiler, mats
Peugeot (406)	√	√					
Rover 2000 and other models							Back shelf/panel storage system
Mitsubishi	√		√			√	
Toyota (Brevis, Harrier, Celsior, Raum)							
SAAB (9S)	√	√					
Volvo (C70, V70)		√					Natural foams,
Vauxhall (Corsa, Astra)				√		√	
Porsche (18 Cayman GT4 Club sport)	√						Body panels, rear wing

**Table 3:** Chemical composition of sawdust.

Plant Source	Classification	Cellulose (%)	Hemicellulose (%)	Lignin (%)	Extractives (%)	Reference
*Hevia* sawdust	Hardwood	39	29	28	4	[133]
Palm Pressed fibre (PPF)	Grass like (neither soft or hardwood)	50.38	16.48	19.46	13.68	[134]
Meranti wood	Hardwood	53.00	18.50	32.4	2.5	[135]
Maple wood	Hardwood	60.15	17.71	11.61	10.53	[136]
Pine wood	Softwood	44	26	26	4	[137]
*Eucolyptus Grandis*	Hardwood	41.6	19.6	27.0	7.3	[138]

**Table 4:** Selective studies on sawdust polymer hybrid composites and other hybrid systems.

Sawdust (SD) Fibre Hybrids	Other Fibre Hybrids	Fabrication and Optimum Effective Fibre Concentration: Sawdust (A) and Other Fibres (B)	Effects of the Fibres Combinations on the Properties of Composites	Recommended Application	References
SD/Banyan/Epoxy	Ramie/Banyan/Epoxy	Sawdust/banyan fibre(s) epoxy hybrid composites were prepared by hand layup process. Better mechanical properties were obtained with ratios: (2% sawdust + 38% banyan) loading whereas the least was observed at (13 sawdust + 37% banyan). Hand layup method was used to fabricate 50% of the fillers (woven mats) and 50% of the polymer matrix. Optimum results were obtained at 37.5 + 12.5 wt.% banyan/ramie fibre composition.	Improved tensile and flexural strength with high energy absorption capacity. SEM images revealed poor adhesion between the fibres and polymer matrix. Increased storage modulus leading to improved stiffness and overall properties. SEM images revealed remarkable bonding between the fibers and epoxy.	Helmets and car interiors	[139,140]
SD/Chicken Feathers/Epoxy	Ceiba Pentandra bark fibre/Chicken feather fiber/Epoxy	The composites were prepared by an extruder. The best results were obtained with a combination of chicken feathers (CF) and sawdust (SD) at a composition, 80H-10CF-10SD. The bioepoxy (BE) composites were prepared by solvent casting and compression moulding. Composition—36.36/9.09/54.54 CPF/C/BE and 18.18/18.18/9.09/54.54 CFF/CPF/C/BE wt.% were studied.	Sawdust and chicken feather enhanced the mechanical properties. The enhancement in mechanical properties was observed through impact, flexural, and tensile strength. Thermal properties were also improved. Better mechanical performance and improved thermal stability for the fabricated composites. Better interfacial interaction between the filler(s) and bioepoxy resin matrix. CPF/C/BE showed better mechanical properties than CFF/CPF/C/BE composites.		[141,170,171]
SD/Cotton/Polypropylene	Jute/Cotton/Polypropylene	Hot pressed hybrid composites fabricated at different fibre loads (between 10 and 20 wt.%). A layer of cotton fabric was spread over the entire cross-section, with a consistent amount for all samples. Optimal performance was observed at 15 and 20 wt.%. Composites were prepared by blending and compression moulding. Better performance of the composites was achieved at 30 wt.% filler loading.	The mechanical properties (Tensile and flexural strength) of the composites improved when reinforced with cotton. The increase in natural filler contents led to a substantial increase in water absorption at 20% fabric/wood-sawdust. Outstanding results in terms of transmission loss and sound absorption in comparison to single fibre reinforced composites. Chemically stable composites.	Acoustic noise reduction field	[143,172,173]
SD/Coir/Epoxy	Sisal/Coir/Epoxy	The epoxy fibre composites were fabricated using the hand layup technique at varying filler and reinforcement loading. The addition of sawdust improved the mechanical properties of the composites up to a certain degree. Higher filler percentage (>5%) increased resistance to resin penetration, rendering specimen redundant. The fibre composites were compression moulded. Better mechanical performance was achieved with the 20/15/65 Sisal/Coir/epoxy composites when the fibres were pre-treated with 5% NaOH.	The addition of filler material improved the tensile, flexural, and hardness properties of the specimen up to a certain percentage, enhancing overall mechanical characteristics. Higher water absorption with the addition of sawdust was observed due to the hydrophilic nature of sawdust and coir. The sisal and coir fibres improved the tensile, flexural and impact properties of natural composites. SEM–hybridization improves reinforcement and adherence of epoxy resin.	Aerospace and automobile industries for their interior parts.	[97,169,174]
SD/Rice husk/Polylactic acid	Banana/Rice husk/Polylactic acid	Fibre reinforced polylactic acid composites were injection moulded at compositions between 2.5 and 10 wt.%), with banana to rice husk ratios of 1:1. Improved performance was observed at maximum loading of 10 wt.% (where 5 wt.% is banana fibre and 5 wt.% is rice husk). Composites of weight percentages ranging from 10 to 40 and banana to rice ratios of 1:1 were prepared by extrusion. Maximum results were obtained with a fiber composition of 40 wt.%.	Significant improvement in their ability to resist deformation but reduced mechanical strength. The fibres improved the overall crystallinity of the polymer by acting as nucleating agents The mechanical properties, i.e., tensile and flexural strength of the composites, were 2.5 times higher. Impact strength also doubled.		[52,137,138,139,140,141,142]
SD/Jute/Epoxy	Oil palm empty fruit bunches (EFB)/Jute/Epoxy	Hand layup in five different composition combinations 80/20. The matrix was kept constant at 20% while the composition of SD ranged from 0 to 40% and Jute 80–40%. Better mechanical performance was observed at 30 wt.% SD loading. Hybrid composites prepared in a stack up mould. The matrix was kept constant at 60% while fibre combination varied from 10 to 40 wt.%. Reduced quantities of the fibres (1:4) led to improved strength and stiffness.	Flexural and impact strength improved. Jute fibres resulted in increased water absorption in comparison to sawdust. Improved flexural, impact, and tensile strength. Morphological analysis showed poor compatibility between the fibres and matrix (cracks, debonding).	Partition walls	[96,175]
SD/Flax/Epoxy	Kevlar/Flax/Epoxy	Sawdust/Flax fibre(s) epoxy hybrid composites were prepared by hand layup technique and studied. Kevlar/flax fiber/epoxy hybrids were created via hand layup and compression moulding. Kevlar at a concentration of 25 wt.% resulted in a twofold increase in the resistance to failure of the composites.	Overall mechanical properties improved. Enhanced tensile, flexural and impact strength. Improved the strength, stiffness, and moisture absorption of flax composites.	Automotive interior, furniture with complex shapes	[51,176]

**Table 5:** Possible pre-treatment for sawdust fibres to improve fibre/matrix interactions for better properties.

Type	Treatment	Short Description	Outcomes of Possible Modifications	References
Chemical	Alkaline	The alkaline reagent is used to clean the surface of plant fibres and modify the cellulose structure. This is known as alkalization, and it reduces the lignin content.	Improved fibre-matrix adhesion, thermal stability, and heat resistivity.	[32,90,111]
	Silane	Involves immersing the natural fibres in a saline solution for a specific duration, allowing the salt to interact with the fibre surface.	Reduce the moisture regain. Improved tensile strength.	[32,90]
	Acetylation	Involves treating fibres with acetic anhydride or acetyl chloride in the presence of a catalyst. The acetylating agent reacts with the hydroxyl groups (-OH) present in the fibre molecules substituting the hydrogen atoms with acetyl groups.	Improve tensile and flexural strength.	[90]
	Peroxide	The peroxides decomposed to form free radicals that react with the hydrogen group of the cellulose fibres and polymer matrix.	Mechanical properties.	[90]
	Benzoylation	Benzoylation is used to decrease the hydrophilic nature of the fibres.	Improve hydrophobicity. The thermal stability of the treated fibres was higher than that of the untreated fibers.	[90]
	Potassium Permanganate (KMnO4)	The chemical reagent potassium permanganate is utilized to change the interfacial contact between the fibre and matrix.	Homogeneity in the mixture, improving morphology contributing to enhanced mechanical properties.	[90]
Subcritical water		Subjecting fibres to a liquid water treatment at temperatures and pressures below its critical point can help soften and swell the fibres, making them more flexible and easier to process.	Good dispersion leading to improved morphology. Enhanced fibre-matrix interactions.	[195,196]
Supercritical water		Supercritical water exhibits unique solvent characteristics at high temperatures and pressures (374 °C and 22.1 MPa), allowing for effective lignin degradation and solubilization. This delignification procedure improves the cleanliness and cellulose content of the fibres, making it more appropriate for a variety of uses.	Improved thermal stability. Reduced moisture content Enhanced adsorption properties.	[197]
Grafting		Grafting is a process of attaching certain chemical moieties or functional groups to the surface of sawdust fibres. This treatment serves multiple functions and can improve the characteristics and performance of the composites.	Improves fibre wettability by hydrophilic matrix materials, allowing for greater fiber dispersion and wetting within the matrix. Improved hydrophilicity can also make interactions with water-based systems easier, such as in biocompatible or biomaterial applications.	[193]
Enzymatic		Enzymes can selectively degrade certain components of lignocellulosic, such as lignin and hemicellulose, while preserving the cellulose structure.	Better dispersion within the matrix (enhanced morphology) Improved mechanical properties.	[198]

**Table 6:** Core factors affecting the morphology of sawdust composites. Adapted from [200,201,202,203,204].

Factors	Influence or Effect
Fibre content (volume fraction of the fibre)	The content or concentration (volume fraction) of sawdust and other reinforcing elements affects the morphology of the composites. Increased reinforcement or filler content can result in more densely packed structures, affecting the arrangement and distribution of the particles. Fibre content optimization is necessary to achieve a balance between processability and better composite qualities (Good results have been obtained between 10 and 40 wt.%).
Fibre orientation	Fibre orientation influences the degree of dispersion within the matrix. Well-aligned and uniformly distributed fibres result in sawdust composites with improved mechanical properties due to enhanced load transfer along the fibre direction. Poor fibre dispersion and alignment, on the other hand, might result in weak interfacial bonding and lower mechanical performance.
Particle size	The particle size of sawdust and other reinforcing materials in the composite can greatly impact the morphology. Smaller particle sizes often result in better particle dispersion within the matrix thus a more homogeneous morphology. Controlling particle size distribution is critical to preventing particle agglomeration or clustering, which can impair the mechanical properties of the composite.
Choice of the matrix	Various matrices and reinforcing agents have different interactions and compatibility, which can affect particle dispersion and the overall morphology of the composite. The matrix choice depends on the desired properties of the composite and the intended application. The matrix or binder must be compatible with the sawdust particles and provide sufficient adhesion and cohesion to form a strong composite. Thermosetting resins (e.g., epoxy, phenolic), thermoplastics (e.g., polypropylene, polyethylene), and bio-based polymers (e.g., starch, lignin) are common matrices for sawdust composites.
Additives	Introducing additives such as plasticizers, coupling agents, or fillers can alter the morphology of sawdust hybrid composites. Their addition can modify the rheological properties of the matrix, encourage interfacial bonding, or improve specific qualities. However, excessive or improper use of the additives may lead to morphological defects, including agglomeration or phase separation.
Fibre/matrix interface(Interfacial bonding)	The binding strength between the sawdust particles and the matrix material is critical for producing the desired shape. Surface treatment of sawdust particles, compatibility of matrix and reinforcing materials, and usage of coupling agents can all affect interfacial bonding. Strong interfacial bonding improves load transfer and the overall mechanical performance of the composite.
Processing techniques and parameters	Different techniques, such as compression moulding, extrusion, or injection moulding have varying effects on particle dispersion and orientation. These methods use varying processing conditions or parameters even when processing the same material. Changes in parameters such as mixing speed, pressure, and temperature can all have an impact on the properties of the composite. Proper control of processing parameters is crucial to achieving the desired morphology.

## Data Availability

The original contributions presented in the study are included in the article, further inquiries can be directed to the corresponding author.
